# Ascorbic Acid and the Brain: Rationale for the Use against Cognitive Decline

**DOI:** 10.3390/nu6041752

**Published:** 2014-04-24

**Authors:** Fiona E. Harrison, Gene L. Bowman, Maria Cristina Polidori

**Affiliations:** 1 Division of Diabetes, Endocrinology and Metabolism, Department of Medicine, Vanderbilt University Medical Center, Nashville, TN 37232, USA; E-Mail: fiona.harrison@vanderbilt.edu; 2 Brain Institute, Department of Neurology, NIA–Aging and Alzheimer’s Disease Center Oregon Health and Science University, Portland, OR 97239, USA; E-Mail: bowmang@ohsu.edu; 3 Geriatrics Department, University of Cologne Medical Faculty, Cologne 50937, Germany

**Keywords:** ascorbic acid, vitamin C, brain, cognitive function, alzheimer’s disease, dementia, aging, elderly, endothelial function, blood-brain barrier, SVCT (sodium-dependent vitamin C transporter)

## Abstract

This review is focused upon the role of ascorbic acid (AA, vitamin C) in the promotion of healthy brain aging. Particular attention is attributed to the biochemistry and neuronal metabolism interface, transport across tissues, animal models that are useful for this area of research, and the human studies that implicate AA in the continuum between normal cognitive aging and age-related cognitive decline up to Alzheimer’s disease. Vascular risk factors and comorbidity relationships with cognitive decline and AA are discussed to facilitate strategies for advancing AA research in the area of brain health and neurodegeneration.

## 1. Introduction

Ascorbic acid (AA; Vitamin C) is a remarkable water-soluble antioxidant concentrated predominately in citrus fruits, strawberries and vegetables (e.g., spinach and broccoli) and found in many supplement formulations (LPI Micronutrient Information Center). The adequate functioning of the human organism in general, and of the brain in particular, is highly dependent on AA, but humans are completely dependent on dietary sources due to the evolutionary process leaving us a gene incapable of producing the enzyme gulonolactone oxidase needed to yield AA from glucose [1]. Thus, humans require a constant stream of AA from the diet and rely on robust “carrier” transport and “barrier” integrity mechanisms to meet the brain’s demand. AA is the most powerful water-soluble antioxidant of the organism, and key to preventing oxidative lipid damage in biological systems [2]. It forms the first line of antioxidant defence under many types of oxidizing conditions. It can rapidly intercept free radicals in the aqueous phase before they attack lipids [3]. As an antioxidant, AA also provides protection against oxidative stress-induced cellular damage by neutralization of lipid hydroperoxyl (LHP) radicals and by protecting proteins from alkylation by electrophilic lipid peroxidation activity [4].

Several pathological processes can involve the production of free radicals, antioxidant depletion, oxidative and nitrosative stress including vascular disease and cognitive impairments seen in aging older adults (Dementia of Alzheimer’s type). Dementia, with its most prevalent form, Alzheimer’s disease (AD), is characterized by an insidious progressive nature that usually begins with memory deficits followed by disturbances in other cognitive domains that eventually reach a level that impacts functions of daily life. Age–related dementias also disrupt the family unit, which must often scramble to meet day-to-day care requirements to compensate for loss of independence. The economic conditions associated with this phenomenon of disability and dependence is an enormous and pressing threat to public health. The major risk factor for dementia and AD remains to be advanced age; therefore the projected impact of AD can be estimated by frequency in strata of the population. For example, in the US about 14% of the older adult population age 65 and older carry a diagnosis of dementia and age 85 and older is about 47%. In either case, these cohort effects will stress the value of anti-AD strategies while general practitioners, geriatricians, neurologists and health care professionals all around the globe are projected to face over 115 million people with dementia from all causes by 2050 [5].

The pathological hallmarks of AD include a loss in synaptic function and accumulation of extracellular amyloid-β plaques and intraneuronal neurofibrillary tangles. Oxidative stress displays chronological primacy in the onset of AD, and in its prephase, mild cognitive impairment (MCI) [6,7,8,9]. Thus, preventive therapy that is safe and effective for reducing oxidative damage seen early in neurodegenerative disorders and applied before the onset of dementia is a public health priority. 

This overview will focus on the biological rationale for the avoidance of AA deficiency in the promotion of healthy brain aging. We include discussion on the biochemistry and transport of AA, much of which has been clarified through basic research using genetically modified mouse models, and also the clinical studies designed to better understand the influence over cognitive health in our aging populations.

## 2. Ascorbic Acid Biochemistry and Transport

AA concentration is higher in the brain than almost all other organs, and in fact may only be equaled in concentration in the adrenal glands. Scurvy, the classical clinical syndrome of AA deficiency, is rare, although not unheard of, in developed populations, but sub-clinical deficiency is still widespread, particularly in at-risk populations such as the elderly, hospitalized and those with poor access to good nutrition. One or more symptoms of scurvy were observed in 18 out of 145 (12%) of elderly patients on admission to hospital, and all but one of this group were classed as being AA deficient as confirmed from plasma levels [10]. Interestingly, 7 of the 23 (30%) controls (no signs of clinical scurvy) also had AA deficiency according to plasma levels, and all of the patients included in the study were classed as having depleted serum AA. Animal studies have shown that brain preferentially retains AA at the expense of other organs with cerebellar, hippocampal and cortical areas appearing to retain AA the most effectively under conditions of depletion which may have a bearing on diseases that target specific areas of the brain [11]. However, the brain is unable to hold maximal or optimal or even sufficient levels in the face of chronic insufficiency, which may have important effects on pathological aging and neurodegenerative diseases. Some populations, such as the elderly and smokers likely require even higher intakes of AA [12]. Roles for AA are widespread and include its vital role as an antioxidant as well as action as a cofactor for a number of dioxygenase enzymes involved in the synthesis of carnitine, collagen, and neurotransmitters including dopamine, norepinephrine and serotonin (reviewed [13]). AA can inhibit LDL oxidation and increase resistance of LDL to oxidation (for review, see [4,14]). AA also plays a role in the function of endothelial nitric oxide synthase (eNOS) by recycling the eNOS cofactor, tetrahydrobiopterin, which is relevant for arterial elasticity and blood pressure regulation [4,15]. Each of these roles plus atherogenic factors may contribute to the preventative role of AA in the development of cognitive impairment.

Evidence of altered glutamate transport (e.g., changes in EAAT2 and EAAT3 transporters) is seen in human AD postmortem samples, particularly in patients with hippocampal sclerosis [16]. AA is released from astrocytes as glutamate is taken up, and this relationship is termed a hetero-exchange although this does not fully represent how the two processes are tethered [17,18]. It is presumed that AA moderates the oxidative stress induced by glutamate [19] and so is protective against overstimulation and cell death. This relationship has been more closely investigated in relation to Huntington’s disease [20,21,22], which also involves cell death. GLT-1 is a high affinity transporter that relies on Na-dependent transport across an electrochemical gradient for rapid removal of glutamate from the synapse. It is sensitive to oxidative stress, and disruption of the transporter leads to glutamate accumulation and hyperstimulation of receptors. Memantine is the first of a new class of drugs for AD that blocks NMDA receptors and its efficacy suggests that further research into glutamatergic signaling and AD is warranted. Therefore, as the role of glutamate transport and NMDA receptors in AD becomes clearer, this may be revealed as another key area where high intracellular AA levels are critical for brain health.

Further excitement has recently been generated in the field of epigenetics with a potential new answer for why AA is concentrated so strongly in CSF and brain parenchyma (reviewed in [23]). Nutrition is perhaps the quintessential example of environmental modification of the genome, and recent work has highlighted a direct role for AA that cannot be replicated with other antioxidants. 5-mc (5-methylcytosine) is oxidized to 5-hmc (5-hydroxymethylcytosine) as part of dynamic DNA demethylation. This reaction, and further oxidation of 5-hmc, are both catalyzed by the activity of TET (ten-eleven translocation) dioxygenase enzymes, for which AA is a critical co-factor (needed for the reduction of iron Fe^3+^ to its active form Fe^2+^) [24,25,26]. Thus AA is vital for neuronal repair as well as new cell generation and here may play a direct role in the transcription and expression of hundreds of different genes. 5-hmc and Tet proteins are abundant in brain and knockout of Tet1 in mice indicated that it may be involved in synaptic plasticity and memory extinction in addition to DNA methylation [27]. The specific relation to AD and other degenerative disorders is not yet clear, but this exciting field may provide new clues.

**Figure 1:**
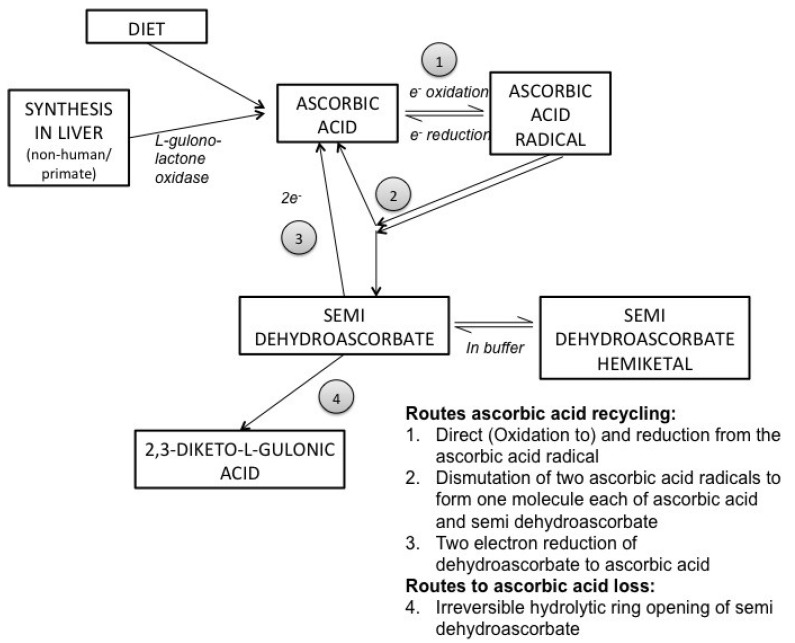
Summary of ascorbic acid oxidation and recycling. Adapted from [13].

AA is a one-electron donor that readily reacts with a range of reactive oxygen species (ROS) to neutralize or decrease their reactivity. Loss of the electron leads to formation of the ascorbate free radical, which can be efficiently recycled to ascorbic acid through enzymatic means (summarized in Figure 1). Ascorbate radicals react preferentially with themselves forming ascorbic acid and dehydroascorbate. This oxidized form of AA can also be recycled back to ascorbic acid, although in some cases it undergoes irreversible ring opening and may be lost. AA also supports the regeneration of other antioxidants, such as vitamin E and glutathione (GSH), in biological tissues, thus combatting oxidative stress through various pathways. This close relationship between GSH and AA is such that AA has often been observed to “take the first hit” for GSH in response to oxidative stressors, and GSH is involved in reduction of dehydroascorbate to ascorbate [28].

It should also be considered that AA and some of its degradation products may be involved in some potentially damaging functions [29]. For example, the Maillard reaction, most commonly studied in relation to food, is also a step in the formation of advanced glycation end products (AGEs). Evidence from a mouse model that selectively over-expressed the vitamin C transporter SVCT2 in the eye [30] implicated AA in age-related damage to crystalline proteins in the lens. There is also some debate as to the potential pro-oxidant role of AA via the Fenton reaction. AA reacts with metal ions in enzymes (e.g., hydroxylases, oxygenases) that require them to be in a reduced state for optimal enzyme function. *In vitro*, reactions with these ions can lead to the production of hydroxyl radicals and other reactive molecules. *In vivo*, catalytic metal ions are less available as their levels are typically kept in check in healthy individuals by metal binding proteins (e.g., ferritin, transferrin). Thus, *in vivo*, the evidence typically supports the antioxidant roles of AA (reviewed in detail [31,32]). One exception may be in the case of iron overload. A limited pro-oxidant effect of increased dietary AA, seen as elevated liver malondialdehyde in combination with decreased glutathione peroxidase, was found in mice fed supplemental dietary iron (although only in the lower and not the high iron diet group) [33]. It should be noted though that this study was performed under conditions of AA sufficiency, in wild-type mice that synthesize their own AA. Such an effect of pro-oxidation reactions was not found in plasma from adults or pre-term infants [3], or in AA- and iron-supplemented guinea pigs [34] and in general AA is not considered a risk factor even in patients with hereditary hemochromatosis [35]. High iron is thought to be a risk factor for AD [36] although the data are equivocal. In a study of 116 AD patients compared to 89 healthy controls, dysregulated iron homeostasis (lower serum iron, ferritin and transferrin, combined with several genetic markers for altered iron metabolism) was associated with AD [37]. The full relationship between AA, iron and AD may warrant further investigation. A further indication of the complexity of the relationships between AA, amyloid and oxidative damage is that in isolated rat brain mitochondria, and in the presence of AA and iron, amyloid-β_1–42_ was actually found to have an antioxidant effect and prevent formation of hydrogen peroxide, presumably through metal chelation [38]. Although aggregated amyloid-β_1–42_ cannot be argued to be healthy in brain tissue, this finding certainly raises interest for researchers in this field who have typically considered amyloid-β to be solely detrimental and an inducer of ROS.

Details of the complexity of the transport of AA within the central nervous system have been understood for almost half a century. Seminal work by Hammarström [39] using radio-labeled AA in guinea pigs first showed that peripherally-administered AA did indeed reach the brain, but that it appeared to travel via the choroid plexus and not traverse the blood brain barrier directly. Reports that AA levels in the CSF exceeded those found in blood, and were less affected by variations in plasma AA, suggested the existence of an active and saturable transporter for AA in the choroid plexus, which was then determined to be the case [40]. The sodium dependent vitamin C transporter 2 (SVCT2) itself was not described until much later but is responsible for transport both at the choroid plexus and in the neurons. There are two sodium-dependent vitamin C transporters, SVCT1 and SVCT2, which are responsible for transport of AA. Distribution of the two transporters varies across organs [41,42,43], but SVCT2 is the only transporter expressed in the brain. Regulation of the active transport of AA by these transporters may also vary across organs and in particular disease states (reviewed [44]). It allows for accumulation of AA in cells against the concentration gradient in SVCT2-dependent tissues. Following characterization of SVCT1 [43,45] single nucleotide polymorphisms (SNPs) were identified in both SVCT1 and SVCT2 and some studies are now being performed to ascertain how they alter AA status and whether they confer additional risk for diseases [46,47]. Several SNPs have been identified in the *SLC23A1* gene coding for SVCT1, and it is argued that this may be less constrained than the *SLC23A2* gene [45,48]. Altered function of the SVCT1 would have important effects on AA absorption and excretion and so would impact nutritional requirements. One synonymous and three nonsynonymous SNPs resulted in diminished vitamin C uptake in *Xenopus laevis* oocytes [49]. One of these SNPs led to an 80% decrease in uptake, and was found to have a relative occurrence of 6%–17% in African-Americans [49]. These studies are of interest to brain research because it is presumed that such SNPs could cause chronically lower circulating AA levels, which would also potentially affect brain levels. Although fewer SVCT2 SNPs have been identified, these could have an even greater effect on brain AA levels and impact disease progression.

**Figure 2:**
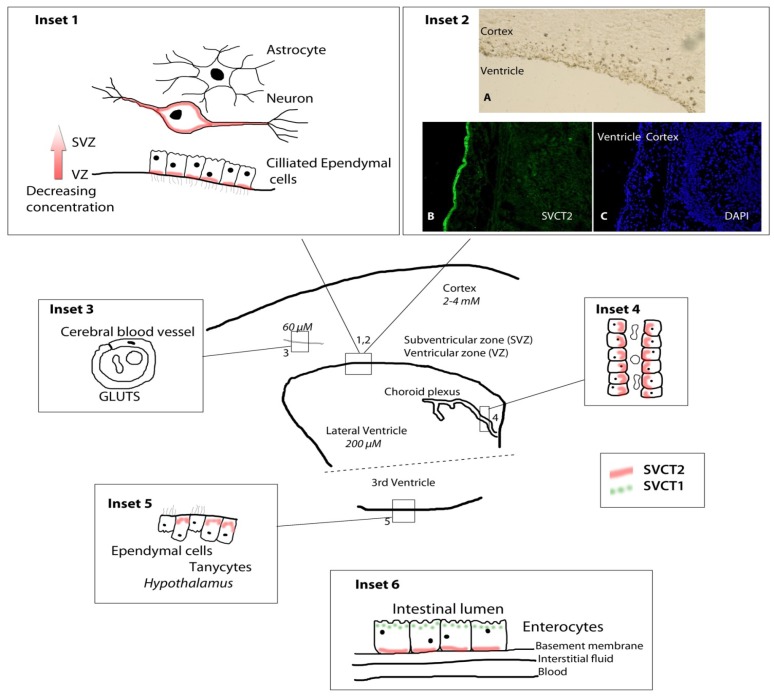
Location and distribution of SVCT1 and SVCT2 transporters and their importance in maintaining optimal brain ascorbic acid levels.

Expression of SVCT2 mRNA and protein varies in brain and other organs during development, presumably directly linked to very high, and changing AA levels during this critical period Nevertheless developmental regulation appears to be particularly specialized and AA regulation of SVCT2 is not common to all cell types, nor to all ages. Thus, SVCT1 and SVCT2 expression, including in brain, is regulated by several disease states associated with oxidative stress (e.g., streptozotocin-induced diabetes and middle cerebral artery occlusion) [50,51] supporting the idea of potential for change in neurodegenerative disorders such as AD. Brain levels of AA in humans are dependent on the SVCT2 (see Figure 2) but also on dietary intake and intestinal absorption via SVCT1. Given that AA can also mediate the permeability of endothelial cell layers in culture [52,53], it can be seen that individual variability in either SVCT1 or SVCT2 transporter function could directly impact brain levels and vasculature in a similar manner to dietary deficiency. One particularly interesting question that may yet be answered with animal studies is the question of how SVCT2 transporter function may change with age and disease status and what impact this would have on AA levels. SVCT2 mRNA and protein both vary during development showing an inverse correlation with brain AA levels, whereas no such changes were seen in brain during AA deficiency in gulo-/- mice indicating that adult brain is unable to respond to long periods of deficiency by altering transport [54,55,56]. Changes in transporter function have not, to our knowledge, been studied in normally aging or AD mouse models, but if transporter function varied among groups, this could lower both CSF and intraneuronal AA levels independently of dietary intake and circulating AA levels.

There are several animals that, like humans, do not synthesize their own AA including guinea pigs, primates and some fish [57]. Nevertheless, transport is conserved across species and study of AA transporters *in situ* can be conducted in most animal models. Cell lines and primary culture techniques are available to study different organs and tissues, which have yielded useful information on expression, membrane location and transport kinetics of SVCT1 and SVCT2 in, for example, intestinal CaCo-2 cells, epithelial cells, endothelial cells, hepatocytes, muscle, intervertebral discs, Schwann cells and others [58,59,60,61,62,63,64,65]. An important consideration is that SVCT2 is regulated to some degree both by AA levels and by oxidative stress and can therefore develop in culture, as seen in astrocytes [66,67], which renders this approach potentially problematic. Other weaknesses of culture systems include the difficulties of maintaining a constant AA level in the media because it is so readily oxidized to dehydroascorbate. Dehydroascorbate can be lost reasonably quickly and can be transported into cells via glucose transporters and then recycled back to ascorbate within the cell. HPLC methods for measuring AA are very accurate if care is taken with sample preparation to minimize loss, and radiolabeled AA is available for assays for quantification in culture. Nevertheless to accurately model human *in vivo* situations, particularly in reference to specific diseases such as AD, animal models are also needed.

Different AA levels across brain regions have been reported in human and rodent brains [11,68,69]. Figure 2 shows a schematic representation of the distribution of SVCT2 (red, solid line) in the brain, and SVCT1 in intestines (green, dotted line). SVCT2 is highly, but not necessarily regularly, distributed throughout the brain. Figure components are not drawn to scale.

**Inset 1**. **Cortex and** **lining of lateral ventricle.** It was originally proposed that AA might follow a pattern of decreasing concentration with increasing distance from the ventricles following a pattern of diffusion [68]. It has since been shown that SVCT2 and AA distribution (indicated by red coloring) depend on cell type and correlate with neuronal density [70,71]. *In situ* hybridization in adult rat showed SVCT2 distribution throughout the brain, with highest expression in olfactory bulbs, hippocampus, cerebellum, cortex and choroid plexus [42]. High SVCT2 expression is seen in the ependymal cells lining the ventricular walls, with decreasing concentration in more external areas of the cortex by *in situ* hybridization and immunohistochemistry techniques [71]; **Neurons.** Primary culture of embryonic (E17) neurons shows SVCT2 in soma and cell processes [71]. Primary culture of mouse hippocampal cultures (E16–17) showed punctate staining of SVCT2 in axons [72]. Using immunohistochemical techniques in hippocampus, SVCT2 was observed in cell bodies but not processes by immunohistochemistry [73]; **Astrocytes **take up dehydroascorbate via glucose transporters (GLUTS) and recycle it to ascorbic acid, which can be taken up by neurons. SVCT2 was not observed in astrocytes by *in situ* hybridization in adult rat brain, even following excitotoxic stimulation with quinolinic acid, although SVCT2 was observed through Northern blot technique in cultured astrocytes from rat embryo [66,74]; **Inset 2. Cortex and lining of lateral ventricle.** Panels A–C show cortex and ventricle. In panel A, AA is stained with silver stain [75] and can be seen at its most concentrated closest to the ventricle lining. In panel B, fluorescent stain for the S19 antibody staining for SVCT2 shows high expression in cells along the ventricle lining. Panel C shows DAPI stain for cell bodies. (Images courtesy of J.M. May); **Inset 3. Cross-section of Cerebral blood vessel.** There is no SVCT2 present in blood vessels in the brain. Glucose transporters (GLUTS) are found in endothelial cells and may transport dehydroascorbate. This contributes a negligible amount to brain AA stores under normal circumstances because there is typically competitive inhibition by glucose. An exception to this state may be after transient ischemia in which case SVCT2 has been shown to develop in mice [76]; **Inset 4.** **Choroid plexus.** SVCT2 is located throughout the choroid plexus for transfer of AA from blood to CSF. High expression of SVCT2 in choroid plexus cells has been shown by *in situ* hybridization in several studies [42,71,74]; **Inset 5. Tanycytes associated with the hypothalamus in the wall of the third ventricle. **Tanycytes are highly specialized hypothalamic glial cells that are capable of forming tight junctions and participate in the formation of the barrier between CSF and brain tissue. SVCT2 has been shown by *in situ* hybridization and immunohistochemistry in different subtypes of tanycytes in mouse brain [75]; **Inset 6. SVCT1 and SVCT2 expression in the intestine**. Expression of both transporters allows for uptake of AA from food and distribution around the body (and to brain). SVCT1 is located on the apical side of the enterocytes and is responsible for taking up AA as it passes through the intestinal lumen. SVCT2 is located on the basolateral side of cells. This is thought to be more important in animals that can synthesize their own AA, but do not consume an AA rich diet, and must still supply enterocytes with AA from blood [60]. Similar localization of SVCT1 on the apical membrane with SVCT2 found on the basolateral membrane is also seen in kidney cells.How AA passes out of the cells into the interstitial fluid and blood supply is still not confirmed, and is a conundrum given the typical direction of the SVCT2 for uptake into cells.

## 3. Ascorbic Acid Transport and Synthesis: Animal Models

Early work on AA and the effects of deficiency was accomplished using guinea pigs that are among the few non-primate mammals that naturally lack a functional *gulo* gene [77]. These studies on the course and effects of scurvy were conducted even before the identification of ascorbic acid as vitamin C [78,79] with inclusion of “the antiscorbutic factor” [80], (such as in fresh raw cabbage). Guinea pigs are particularly sensitive to AA (and other) deficiencies [81,82], and are still a highly utilized model for a number of human disease states, for example in relation to dyslipidemia (reviewed in [83]). The recent (since 2000) increased focus on mouse models results from the ability to use genetically altered mouse lines to impact both transport and synthesis in the same animals. Much more is known about the mouse genome than guinea pig and in many cases the models have been also bred to the same background strain making experiments directly comparable. Data can thus be interpreted, and experiments planned, with reference to the huge literature of biomedical and pharmaceutical research that has already taken place in mice making disease and treatment-relevant findings much more likely. Brief descriptions of the relevant mouse models, including gulo knockout mice, and SVCT1/2 knockout models and SVCT2 transgenic mice are reported hereafter.

Gulo knockout mice: Gulo knockout mice carry an inactivated form of the gene l-gulono-γ-lactone oxidase and are thus completely dependent on dietary AA [84]. These mice, when maintained on sub-optimal supplementation levels, are the closest to the human state of lifetime AA dietary deficiency. Knockout mice fed low levels of AA have decreased antioxidant capacity and also altered cholesterol metabolism and exhibit profound changes in vasculature. Oxidative stress has since been confirmed in these mice in adults and during development [11,85,86]. These mice also have an aberrant behavioral phenotype including poorer sensorimotor skills, and altered response to activity-inducing effects of cholinergic and dopaminergic compounds scopolamine and methamphetamine even when adequately supplemented as adults [86,87,88]. It is proposed that these changes result from low AA during some part of the developmental process. AA deficient gulo mice have been the subject of metabolomics profiling which has identified metabolic shifts as a response to oxidative stress of AA deficiency (e.g., upregulation of glutathione production, decreased carnitine production) [89]. Neutrophils from AA deficient gulo mice are more likely to undergo necrosis than normal apoptosis. They are not recognized by macrophages and thus clearance is also affected [90]. Such responses are important in any disease where inflammation is a factor, such as AD. Senescence marker protein 30 (SMP30) was first identified as a protein that decreased with age. Sequencing and creation of an SMP30 knockout mouse later identified this protein as the gulo enzyme [91,92]. These mice are likewise dependent on dietary AA and under conditions of deficiency their brains are susceptible to oxidative stress (generation of superoxide) [93,94]. A further mouse line exhibiting spontaneous bone fracture derived from in-bred balb/c mice [95] does not synthesize AA [96]. These mice die at an early age if not supplemented, and have been used for studies of AA level and gene transcription [97], however, this line is much less well-described. Disruption to catecholamine levels in brain and adrenal glands has been shown in each of these mouse lines [98,99]. Such data that follow a consistent pattern across the different mouse models are a strong support for the role of AA in any particular area and also of the validity of the models themselves.

**Figure 3:**
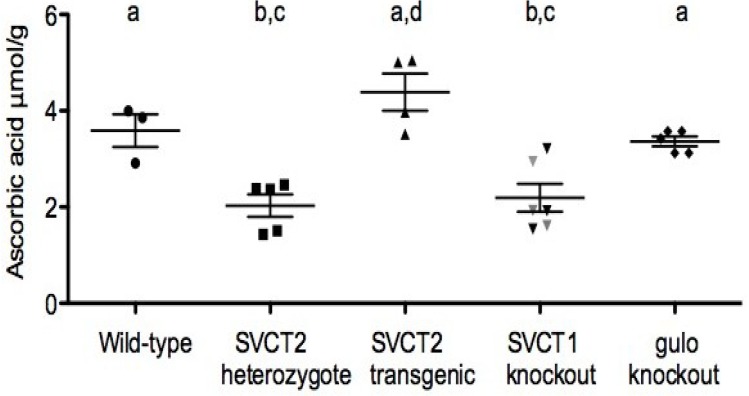
Direct comparison of cortex levels of ascorbic acid in different mouse lines run concurrently. All mice used were male, aged 9–12 months (retired breeders).

SVCT1 knockout mice: SVCT1 knockout mice are viable and fertile [49]. They excrete significantly more AA in the urine than wild type and heterozygous littermates and have lower circulating blood levels. In addition to this loss of renal reabsorption of AA, uptake into liver was also dramatically decreased. Also of interest is the lower brain levels in these mice, presumably linked to lower circulating levels because SVCT1 is not expressed in brain and so should not have a direct effect (Figure 3).

SVCT2 knockout mice: The first report of the SVCT2 knockout mouse described homozygous mice that did not survive past birth (newborns never take a first breath) and had almost undetectable AA levels in all organs measured (brain, liver, adrenal glands, kidney, pituitary glands, pancreas and muscle) [100]. Subsequent studies found that AA levels were also decreased in placenta and lung as well as brain and other organs reported previously [101]. These low AA levels were associated with increased oxidative stress and cell death. Hemorrhaging, initially only reported in cortical areas [100] was also seen in the brain stem area which may contribute to the lack of breathing in the newborn mice, particularly because no obvious problems were identified in the lung in the earlier study. Primary cultured cells from hippocampi of embryonic SVCT2 knockout mice showed decreased neuronal activity, stunted neurite outgrowth and were much more sensitive to exogenously administered oxidative stress [72]. Investigation into catecholamine systems in these mice has yielded mixed data. One report showed large decreases (50%) of norepinephrine in the adrenal glands with no change in dopamine levels, whereas smaller changes in the brain (decrease of 20%–25%) were not significant for either dopamine or norepinephrine in SVCT2 knockout mice [102]. Additionally, morphological differences in the chromaffin cells of the adrenal glands were also noted in this study. Other reports have shown significant alteration to dopamine and norepinephrine systems, including metabolites, in embryonic SVCT2 knockout and SVCT2 transgenic (described below) brains [103].

Increase in AA in SVCT2-Tg mice is dependent on level of over-expression, which varies among mice and in this small sample was only 1.3-2.0-fold compared to wild-type (not shown). Brain levels did not differ between supplemented (black triangles) and non-supplemented (grey triangles) SVCT1 knockout mice so combined average is shown. Normal brain levels can be achieved in gulo knockout mice with supplementation of at least 0.33 g/L, (shown here at supplementation with 1.0 g/L). Mean AA levels that share a superscript do not differ. Differing superscripts indicate significant difference ^(a, b)^ *p* < 0.05, ^(c, d)^ *p* < 0.001 by Univariate ANOVA with Tukey *post-hoc* comparisons (overall group effect F (4,18) = 13.0, *p* < 0.001).

SVCT2 transgenic mice: Recently, a new mouse line has been created that expresses additional copies of the SVCT2 [104]. This mouse has increased SVCT2 mRNA expression in all organs measured and a related increase in AA level in all organs except the liver, with up to two-fold increases in the brain, depending on mRNA expression. These mice were otherwise phenotypically indistinguishable from wild type but were more resistant to a severe oxidative stress in lung induced by paraquat. Although not designed to represent an attainable human situation, these mice may be able to inform on SVCT2 regulation and the role of vitamin C in high oxidative stress situations such as neurodegenerative disease.

## 4. Relation of Mice Models to Human Studies

A number of shortcomings exist to studies of AA in humans, such as accurate assessment of intake and determination of tissue levels of AA (discussed below). Thus, information from animal models can be critical to understanding changes in the brains of humans under AA deficiency. The gulo and SVCT2 knockout models have already been used to model long-term AA deficiency in disease states such as Alzheimer’s disease, diabetes and atherosclerosis [88,105,106,107] by crossing them with other mouse models of disease (e.g., APP/PSEN1, ApoE). These models can even be crossed together to create *in vivo* models of extreme deficiency [108], although these may be less directly applicable to the human case. One key advantage to such models is the ability to investigate numerous biochemical correlates to disease and nutritional deficiency, including behavioral and cognition changes. Of course there are weaknesses to any animal model, particularly when genetic changes are artificially manufactured, and follow an all-or-none approach rather than the gradations of functional change from mutations seen in the general population. Nevertheless, the availability of models to study all aspects of transport and synthesis together provides a very strong future for pre-clinical AA research. It is important to note, however, that the changes reported in these models, particularly oxidative damage, or changes in neurotransmitter function, are not specific to Alzheimer’s disease, and in fact are relevant to many types of neurodegenerative diseases, and to an extent in normal as well as pathological aging. For example, none of these mouse models exhibits robust cognitive deficits from AA deficiency alone. Future research must identify exact roles for AA in disease pathology, and how this can be applied specifically to Alzheimer’s disease process as well as other disease states where applicable. This includes studying the impact of chronic deficiency resulting in sub-optimal brain levels, without clinical signs associated with scurvy. Knowledge of how AA transport and accumulation is regulated in specific brain areas, and how this may be affected by specific SNPs or disease states may permit better understanding of when and how best to intervene to correct levels, or how to identify populations that may be more at risk of deficiency.

## 5. Dietary Intake of Ascorbic Acid, Cognition and Alzheimer’s Disease

Associations between risk for AD and AA intake have been investigated in several large population studies, both in the US and also in one large European sample. One early study appeared very promising when data was reported from the Chicago Healthy Aging Project (CHAP) showing that none of the 633 > 65 years old, dementia-free participants that supplemented with AA, developed AD over the follow up period (mean 4 years) [109]. Study of dietary intakes in the same cohort did not support the same protective effect of AA [110]. It may be that even the dietary levels ingested were insufficient for the protective effect and that supplements are necessary to maintain optimal levels. In the earlier study only supplementation was considered, as single nutrient, multi-vitamin or no supplement, without comment as to dose. In the latter study, while the median all-source intake was 124.7 mg/day, with 16.1% of population taking supplements of some kind, the intake ranges were very large. In the lowest quintile intake from food and supplements was estimated at below 93 mg/day and the highest quintile was between 310 and 2530 mg/day. In neither case are blood AA levels reported and thus it is difficult to accurately determine AA status in the different populations. The differences between dietary and synthetic AA intake, and their comparable bioavailability in humans, are discussed in this issue [111]. Self-reported AA intakes (alone or in combination with vitamin E) were not predictive of AD diagnosis in a cohort of nearly 5,000 participants aged 65 and older over the course of 5 years [112]. Nor were beneficial effects of AA intake observed in a study of 980 dementia-free men and women of the Washington Heights-Inwood Columbia Aging Project [113].

A more complex pattern of effects was reported in the Honolulu Asia Aging Study which comprised men aged 71 to 93 years [114]. In cognitively intact individuals, AA intake was associated with a higher likelihood for enhanced cognitive function. High AA and vitamin E intake were associated with lower likelihood for vascular dementia. In contrast, there was no relationship between AD diagnoses and AA intake. Findings from the Cache County Study [115] suggest that AA and vitamin E supplementation may have some synergistic effects in reducing risk for AD, but AA alone did not decrease risk for AD. Self reported intake questionnaires and telephone assessments of cognitive ability were used in the very large cohort of nearly 15,000 women in the Nurses health study. No consistent associations were found between AA and cognitive ability in this group [116,117].

Perhaps one of the most interesting results came from the Rotterdam Scan Study [118]. This large study of over 5000 participants reported an 18% reduced relative risk for AD with higher AA intake. Most revealing was that the most dramatic protective effects were seen in smokers. Smoking leads to rapid depletion of AA in plasma in addition to additional ROS generated following inhalation of smoke. It therefore seems likely that rescuing AA deficiency in this group may have been more useful than supplementing AA on top of dietary in healthier individuals.

If AA deficiency really is a key factor in the development of AD, then it might be expected that populations with poorer intake would be more prone to developing dementia, or follow a faster path of deterioration once diagnosed. For the most part these studies draw strength from the large sample sizes used, numbering in the thousands. On the other hand, reliance on dietary intake questionnaires may be problematic, particularly in a study of cognitive ability where reliability may be acutely affected by even mild changes in recall ability [119]. Current intake may not reflect lifetime dietary habits and given data that suggest that amyloid plaque burden begins to form well before middle age [120], intakes during younger adulthood may be equally as important as supplements taken by older adults, perhaps contributing to a biological buffer against disease pathogenesis. Overall, the two promising positive results are outweighed by the seven studies that did not confirm a link between AA and AD. Ideally, reliable biomarkers of diet should be employed instead, or preferably in addition to study of dietary intake [121]. Measurement of AA levels, preferably over several time points, permits determination of whether participants have deficient or depleted AA levels, and more importantly allow grouping according to AA level. In this way dietary *versus* supplemental intake is not as important as the resulting AA levels, and high level supplements, which, due to the limitations of the SVCT1 transporter in the intestines, may not functionally provide more AA than lower supplements or good diet, are not weighted more heavily and biasing results.

## 6. Blood Ascorbic Acid, Cognition and Alzheimer’s Disease

For studies that have made measurements of biological AA levels in blood and/or CSF, the sample sizes are typically much lower than the population based studies described above (see Table 1). The logistics and cost of so many measurements may be prohibitive, and even in studies that have a prospective aspect, true control groups are not possible. It would be unethical to maintain one group of participants at low AA levels, and it is also not possible to control for lifetime dietary habits in these studies. Nevertheless, these cross sectional studies can certainly provide an accurate and useful picture of typical nutrition profiles in various populations, alongside cognitive analyses.

**Table 1:** Observational Studies of Ascorbic Acid Status in Dementia and Non-Impaired Elders (NIE) adapted from [122].

Authors	Sample	Design and Methods	Conclusion	Association y/n
Goodwin, 1983 [123]	260 dementia-free men/women Age ≥ 60 year Community-dwelling	Cross-sectional Plasma, 3 day diet recall Halstead-Reitan Categories Test, WMS-R delayed	Plasma AA associated with higher verbal memory; elders in the 90th percentile of plasma AA had better calculating ability and delayed recall	y
Gale, 1996 [124]	921 men/women Age ≥ 65 year English-Scottish-Welsh	Cross-sectional Plasma Hodkinson test	Plasma AA ≤ 11.91 µM was associated with 1.6 higher odds of cognitive impairment (95% CI: 1.1–2.3). Less than 27 mg of AA intake per day was associated with 1.7 higher odds for impairment	y
Riviere, 1998 [125]	19 ctls MMSE 24–30 20 severe AD MMSE 0–9 24 mod AD MMSE 10–23	Cross-sectional Plasma Mini-nutritional assessment	Plasma AA was incrementally lower by degree of cognitive impairment in AD, this observation was not explained by lower intake of AA	y
Charlton, 2004 [126]	93 men/women 50 ctls 43 dementia Age ≥ 65	Cross-sectional Plasma Mini-nutritional assessment	Plasma AA was lower in subjects with dementia compared to controls, this observation was not explained by lower intake of AA	y
Polidori, 2002 [127]	75 women 40 ctls (85.4 ± 4.4 year) 35 AD (85.9 ± 5.5 y) Age-matched Mean age 85 year	Cross-sectional Plasma NINCDS-ADRDA criteria	Plasma AA was lower in AD than controls (18 ± 6 µM *vs.* 36 ± 6, *p* < 0.001) AD subjects had increased plasma lipid peroxidation and less resistance to peroxyl-radical	y
Polidori, 2004 [128]	141 55 ctls 63 AD 23 VD	Cross-sectional Plasma	Plasma AA was lower in AD and VD *versus* NIE, no differences between AD and VD	y
Perrig, 1997 [129]	442 men/women (*n* = 312/132) Community-dwelling Age ≥ 65 (mean 75)	Prospective, cross-sectional Plasma Memory, WAIS-R vocabulary test	Plasma AA in 1971 associated with better cognitive performance in 1993; plasma AA associated with better free recall, recognition, and vocabulary, but not priming and working-memory in cross-sectional analysis	y

AD, Alzheimer’s disease; VD, vascular dementia; Ctls, control subjects; NIE, non-impaired elders; AA, ascorbic acid; MMSE, mini-mental state exam (Folstein); NINCDS-ADRDA, National Institute of Neurological and Communicative Disorders and Stroke and the Alzheimer’s Disease and Related Disorders Association; WAIS-R, Wechsler Adult Intelligence Scale—revised.

An early study by Goodwin *et al.* [123] used the revealing method of dividing participants into percentiles according to AA status. Using these methods verbal memory recall and calculations were indeed associated with higher plasma AA. In contrast, and in support of the idea that deficiency can impact development of AD and other dementias Gale *et al.* [124] report a significantly increased risk for cognitive impairment in those with depleted (<12 µM) plasma AA or very low intake (<28 mg/day). Higher plasma AA was associated with better free recall, recognition and vocabulary, but not working memory in a prospective sample of older men (>65 years) [129]. In general, plasma levels of AA in the above studies have been consistently observed to be around 20 µM in patients with AD, *i.e.*, about the half of those measured in controls [7,125,127]. In agreement with studies showing that plasma AA levels are depleted in AD independent of dietary intake, peripheral AA depletion in AD patients with respect to controls has been repeatedly confirmed after correction for age, gender, fruit and vegetable intake, and comorbidities [7,125,127]. Another element strongly suggestive of low AA levels as a co-causal factor for neurodegeneration and AD rather than epiphenomenon of AD is the observation of similarly depleted plasma AA levels in both MCI patients and AD patients compared to controls [7]. Comparison of all these studies where effects are on particular subtypes of memory of cognition, *versus* disease risk, may indicate that avoiding deficiency, and optimally supplementing with AA may benefit different facets of cognitive health. Nevertheless, the directions of effect seem to be in the same direction.

## 7. Cerebrospinal Fluid Ascorbic Acid, Cognition, and Alzheimer’s Disease

Examination of the AA in the CSF reflects nutrient content with direct access to the brain parenchyma. This proximal representation should be considered the gold standard of brain nutrition in living subjects [130]. Presumably owing to the more intrusive nature of the testing far fewer studies have reported CSF AA (Table 2).

Paraskevas *et al.* [131] report high variation in plasma AA levels in hospitalized groups of 17 AD, 19 amyotrophic lateral sclerosis and 15 control patients, but reasonably stable CSF levels. They conclude that maintenance of the plasma:CSF ratio must be due to appropriate action of the SVCT2 at the choroid plexus. Lack of data on dietary intakes and case classifications limit the full utility of this study. Quinn *et al*. [132] examined the cross-sectional differences in CSF and plasma AA between 10 AD cases *versus* 10 healthy controls. Another cross-sectional study conducted by Glaso *et al*. [133] examined the mean differences between plasma and CSF AA in women with and without dementia of AD type. In both of these studies mean plasma and CSF AA were less (although not significantly in [132]) and the CSF-to-plasma AA ratio was higher in AD *versus* controls. 

**Table 2:** Observational studies of CSF Ascorbic Acid, Cognitive Function, and Alzheimer’s disease * adapted from [122].

Authors	Sample & Design	Plasma, µM		CSF, µM		CSF:Plasma Ratio		Conclusion
		Ctls	AD	Ctls	AD	Ctls	AD	
Paraskevas, 1997 [131]	32 15 ctls (58 ± 12 year) 17 AD (62 ± 7 year) Men/women Age-gender matched Cross-sectional	43 ± 13	45 ± 19	166 ± 45	156 ± 38	3.6 ± 0.5	4.1 ± 1.6	No differences between AD and controls
Quinn, 2003 [132]	20 10 Ctls (MMSE 29 ± 1), 10 AD (MMSE 19 ± 7) Age-gender matched Mean age 66 y Cross-sectional	86 ± 39	58 ± 42	238 ± 48	207 ± 64	3.1 ± 1.1	5.0 ± 2.6	CSF: plasma AA ratio higher in AD (*p* = 0.048). Mean plasma and CSF AA were lower in AD, but not significantly.
Glaso, 2004 [133]	38 18 Ctls (MMSE 27), 20 AD (MMSE 16) Women Age-matched (75–85 year) Cross- sectional	80 ± 28	44 ± 25	167 ± 23	140 ± 37	2.1 ± 0.7	3.3 ± 1.4	CSF: serum AA ratio higher in AD *versus* controls (*p* = 0.001) but both plasma (*p* = 0.002) and CSF (*p* = 0.038) AA were lower in AD. Subjects were well-nourished and without vascular disease
Bowman, 2009 [134]	32 AD (MMSE 19 ± 5) Men/women Mean age 71 year Prospective		41 ± 30		129 ± 52		4.0 ± 1.6	Neither plasma nor CSF AA was predictive, but CSF: plasma AA ratio associated with slower cognitive decline over 1 year (age, gender, education, apoEe4, and cognitive function at baseline adjusted *p* = 0.025). Interaction between CSF AA Ratio and BBB integrity suggesting AA leaking from brain to periphery
Arlt 2012 [135]	23 AD men/women, already taking cholinesterase inhibitors. Randomized to AA (2 × 500 mg/day) and vitamin E (400 IU/day) (Age 67.7 ± 7.2, baseline MMSE 20.0 ± 5.3)		Baseline 201.4 ± 25.9, 1 month 229.9 ± 36.7, 12 months 233.6 ± 44.8		Not measured			No direct effect of antioxidants on performance, however supplements did increase AA and vitamin E in CSF, with antioxidant effect. Greater oxidation of CSF lipids was associated with faster decline of cognitive ability
	No supplements (Age 73.7 ± 5.3, baseline MMSE 21.7 ± 5.5)		Not measured		Not measured			
Galasko 2012 [136]	16 weeks with 500 mg/day AA, plus 800 IU alpha-tocopherol and 900 mg/day alpha-lipoic acid in subjects with mild to moderate AD		Not measured		Not measured			Measures of CSF oxidative stress were decreased, but amyloid and tau markers were not. Nor were there any beneficial effects on cognitive decline.

* Total of 79 AD and 43 controls with CSF AA analysis; Ctls, control subjects; AD, Alzheimer’s disease subjects; MMSE, mini-mental state exam.

Ideally, such studies would be prospective, with baseline and later AA levels taken of both plasma and CSF AA across at least one year, in addition to cognitive assessment. One such prospective analysis also included CSF Albumin Index to reflect blood-brain barrier integrity in living probable mild to moderate AD patients over a year [134]. However, a higher CSF-to-plasma AA ratio was associated with a slower rate of decline although neither plasma nor CSF AA alone was predictive. The relationship between CSF AA ratio and rate of decline was attenuated when CSF Albumin Index (a validated measure of BBB integrity) was added to the regression model. These findings suggest that maintenance of high CSF-to-plasma AA ratio may be important to preventing cognitive decline in AD and that BBB impairment unfavorably alters this ratio. This study was unable to distinguish whether transport mechanisms for AA (*i.e*., SVCT2) were disturbed as well as the integrity of the BBB since the CSF albumin index reflects only barrier disturbances to our knowledge. How much of this “barrier” impairment is accompanied by “carrier” dysfunction is one area for future research interest.

A recent study on dietary supplements examined the antioxidant effect of 1000 mg AA per day (two × 500 mg) plus 400 IU vitamin E in mild to moderate AD patients who were also taking cholinesterase inhibitors [135]. In this relatively small, open label study, one year of supplements did not have a direct effect on cognition. Nevertheless, the authors were able to successfully demonstrate that supplements led to higher AA and vitamin E in the CSF and also decreased lipid peroxidation in the CSF. Greater levels of oxidation were also associated with faster cognitive decline. Unfortunately CSF was only measured in the supplement group, and dietary intake, or baseline group differences were not accounted for (although none of the subjects was taking supplements at the start of the study). In an additional study supplementation for 16 weeks with 500 mg/d AA with 800 IU/d vitamin E and 900 mg/day alpha-lipoic acid in 24 mild to moderately affected AD patients screened to exclude cases of vascular disease, were compared to 18 controls [136]. The antioxidant mix was also found to decrease oxidative stress in the CSF (F_2_-isoprostanes), however, no effects were seen on CSF Aβ_1-42_, tau or *p*-tau. No improvements were seen in cognition, in fact this group appeared to suffer faster cognitive decline. Critically, all subjects in the study were allowed to continue taking their own vitamin supplements up to 200 mg/day AA, this included 52% of the antioxidant group and 43% of controls. AA levels at baseline or following treatment are not reported, and thus it is not possible to assess whether groups truly differ; 200 mg/day in a supplement plus a reasonable diet could permit AA repletion in the placebo group and mitigate the chance of seeing differences in cognition although the oxidative stress data clearly indicates benefits of the antioxidant cocktail. A recent review of several studies above concluded that CSF levels within normal range for AA (and folate and additional CSF proteins), despite lower plasma levels indicated preservation of choroid plexus function and AA transport into CSF [137]. However, they also discuss the lack of definitive data on potential for change in CSF volume or turnover.

## 8. Ascorbic Acid and Vascular Cognitive Aging

The primary focus of this review is on AD, both with and without a vascular component. The main pathological hallmarks of AD (amyloid and tau deposits, neuronal death, neurotransmitter signaling, synaptic density) are shared across cases because the nature of AD is multifactorial. However, in the same way that many of these symptoms are also found in other cases of dementia, e.g., pure vascular dementia, frontotemporal dementia, dementia with lewy bodies, it can easily be argued that many of the findings described are related to neurodegeneration in general and are thus applicable to many dementias and even normal aging. Previous schools of thought have considered vascular- *versus* AD-type dementia cases as separate entities, however accumulating evidence suggests that vascular pathology plays a central role in AD onset and development [138,139,140]. In a community sample of older adults (75+ years) almost 50% of the clinically diagnosed AD cases showed a possible vascular component [141]. Similarly, the next most prevalent dementia form, vascular dementia, presents overlapping traditional hallmarks of AD including amyloid-β accumulation. As a direct consequence of this complexity, a cure is difficult to find, and prevention becomes even more critical. The role of AA has recently been reviewed in relation to endothelial cell function, which may explain importance in its role in vascular health. In addition to the more well-known functions of AA such as synthesis and deposition of collagen in the basement membrane and antioxidant defense against ROS, other roles include stimulating endothelial proliferation, inhibiting apoptosis, and sparing endothelial cell-derived nitric oxide to help modulate blood flow [15].

The ability of AA to prevent age-associated cognitive decline and dementia risk may require a population with high vascular risk. AA has demonstrated some ability to reduce vascular risk factors and more recently vascular comorbidities are being acknowledged as important factors to reduce for prevention of age related dementias [138,139,142,143,144,145,146,147]. Some of the “vascular” mechanisms include: (1) reducing carotid intima-media-thickness [2,148]; (2) reducing lipid peroxidation [3,4,149,150,151]; and (3) reducing endothelial dysfunction [15].

We recently observed healthy elderly in the lowest plasma AA tertile at higher risk of carotid intima-media thickness >1.2 mm; a threshold established as pathologic by the European Society of Hypertension and the European Society of Cardiology 2007. It should be noted that this relationship was not appreciated with other antioxidants that include: uric acid, vitamins A and E, and enzymatic enzymes superoxide dismutase and glutathione oxidase activity. Another study of 8,453 participants in NHANES II concluded that individuals with plasma AA ≥ 45.4 µM had a 21-25% reduced risk for CVD-related death and a 25-29% reduced risk of all-cause mortality compared to the participants with plasma AA < 23 µM [152]. In the European Prospective Investigation into Cancer and Nutrition (EPIC)-Norfolk study [153] a 33% lower risk of developing coronary heart disease was shown in subjects with the highest plasma AA compared to the lowest (mean, 27.6 µM) over six years of follow up. Long in advance to these results, the EPIC-Norfolk study had found that plasma AA concentrations in 8860 men and 10,636 women were inversely correlated to mortality from all causes and CVD. In this study, each 20 µmol/liter increase in plasma AA was associated with a 20%–30% reduction in risk for all-cause and CVD mortality [154]. When 563 elderly men were randomly allocated to one of four treatment groups in a clinical trial that included dietary intervention, omega-3 supplementation, both or neither, carotid intima-media thickness progression over a three-year term was reduced in those undergoing dietary intervention that included daily AA intake [155]. Vitamin E [156] or combined antioxidants [157] in the dietary intervention groups were not successful.

These data suggest that AA has a role in modifying vascular risk factors and vascular disease, which could represent mechanisms by which AA might reduce dementia risk in people carrying this vascular risk profile [158].

Recent research has demonstrated that it is now possible to non-invasively measure AA (and GSH) levels in the human brain using MEGA-PRESS MRI (a type of spectroscopic MRI imaging) [159]. These techniques were used to compare AA, GSH and lactate in brain in 22 young (20 years) *versus* 22 normally aging (76.6 years) subjects [160]. The study reported decreased GSH and increased lactate with age, indicating oxidative damage, but no change in AA between the groups. The authors recruited candidates that ate less than five fruits and vegetables per day and did not take supplements, then provided food calculated to contain 30 mg/1000 kcal in an attempt to provide all subjects with the recommended daily intake. Thus, potential group differences were already minimized, and at ~60 mg/day circulating and brain AA levels may still have been sub-optimal in all subjects, reflected by the estimated brain levels of 0.4–1.2 μmol/g (their Figure 3 [160]) tissue wet weight. It is reported that brain AA content was not associated with AA in blood, however these data are not recorded. So although this study does not provide a definitive statement of AA in brain in the elderly, development of this fascinating and ingenious technique to measure AA in brain may add a critical factor to future studies of the role of AA in the brain.

## 9. Conclusion

This review highlights several key points relating to the role of AA in healthy brain aging: (1) both human and animal studies demonstrate AA deficiency in association with oxidative stress markers, and oxidative stress is a consistent observation in AD; (2) there is inconsistency among the large observational studies relating dietary intake of AA to cognition. However, it remains unclear whether this inconsistency is methodological in nature (e.g., the subjective dietary surveys used to capture AA intake) since biomarkers of both AA (and oxidative stress) present more consistent results favouring an important role for AA in cognitive health; (3) there are genetic (SVCT1 and SVCT2 SNPs) and non-genetic (e.g., age) factors that modulate AA absorption and assimilation, which could indicate an increased demand for AA in subsets of the population such as the elderly and those with an AD diagnosis. Thus, we do not suggest that AA deficiency in isolation can explain AD neuro- and psycho-pathology, however, we do propose more research focused on investigating the specific role of AA in AD pathogenesis with meticulous attention to the study design (e.g., people with low AA and high vascular risk may be best suited for intervention). This activity should provide more conclusive data on this remarkable micronutrient highly concentrated in the brain.

## References

[1] Nishikimi M., Fukuyama R., Minoshima S., Yagi K. (1994). Cloning and chromosomal mapping of the human nonfunctional gene for l-gulono-gamma-lactone oxidase, the enzyme for l-ascorbic acid biosynthesis missing in man. J. Biol. Chem..

[2] Frei B., England L., Ames B.N. (1989). Ascorbate is an outstanding antioxidant in human blood plasma. Proc. Natl. Acad. Sci. USA.

[3] Berger T.M., Polidori M.C., Dabbagh A., Evans P.J., Halliwell B., Morrow J.D., Roberts L.J., Frei B. (1997). Antioxidant activity of vitamin C in iron-overloaded human plasma. J. Biol. Chem..

[4] Traber M.G., Stevens J.F. (1997). Vitamins C and E: Beneficial effects from a mechanistic perspective. Free Radic. Biol. Med..

[5] Prince M., Bryce R., Albanese E., Wimo A., Ribeiro W., Ferri C.P. (2013). The global prevalence of dementia: A systematic review and metaanalysis. Alzheimer’s Dement..

[6] Pratico D., Clark C.M., Liun F., Rokach J., Lee V.Y., Trojanowski J.Q. (2002). Increase of brain oxidative stress in mild cognitive impairment: A possible predictor of Alzheimer disease. Arch. Neurol..

[7] Rinaldi P., Polidori M.C., Metastasio A., Mariani E., Mattioli P., Cherubini A., Catani M., Cecchetti R., Senin U., Mecocci P. (2003). Plasma antioxidants are similarly depleted in mild cognitive impairment and in Alzheimer’s disease. Neurobiol. Aging.

[8] Mecocci P., Polidori M.C. (2012). Antioxidant clinical trials in mild cognitive impairment and Alzheimer’s disease. Biochim. Biophys. Acta.

[9] Mangialasche F., Kivipelto M., Mecocci P., Rizzuto D., Palmer K., Winblad B., Fratiglioni L. (2010). High plasma levels of vitamin E forms and reduced Alzheimer’s disease risk in advanced age. J. Alzheimer’s Dis. (JAD).

[10] Raynaud-Simon A., Cohen-Bittan J., Gouronnec A., Pautas E., Senet P., Verny M., Boddaert J. (2010). Scurvy in hospitalized elderly patients. J. Nutr. Health Aging.

[11] Harrison F.E., Green R.J., Dawes S.M., May J.M. (2010). Vitamin C distribution and retention in the mouse brain. Brain Res..

[12] Brubacher D., Moser U., Jordan P. (2000). Vitamin C concentrations in plasma as a function of intake: A meta-analysis. Int. J. Vitam Nutr. Res..

[13] Harrison F.E., May J.M. (2009). Vitamin C function in the brain: vital role of the ascorbate transporter SVCT2. Free Radic Biol. Med..

[14] Kaliora A.C., Dedoussis G.V., Schmidt H. (2006). Dietary antioxidants in preventing atherogenesis. Atherosclerosis.

[15] May J.M., Harrison F.E. (2013). Role of Vitamin C in the Function of the Vascular Endothelium. Antioxid. Redox Signal..

[16] Proper E.A., Hoogland G., Kappen S.M., Jansen G.H., Rensen M.G., Schrama L.H., van Veelen C.W., van Rijen P.C., van Nieuwenhuizen O., Gispen W.H. (2002). Distribution of glutamate transporters in the hippocampus of patients with pharmaco-resistant temporal lobe epilepsy. Brain.

[17] Wilson J.X., Peters C.E., Sitar S.M., Daoust P., Gelb A.W. (2000). Glutamate stimulates ascorbate transport by astrocytes. Brain Res..

[18] Estrada-Sanchez A.M., Rebec G.V. (2012). Corticostriatal dysfunction and glutamate transporter 1 (GLT1) in Huntington's disease: Interactions between neurons and astrocytes. Basal Ganglia.

[19] Ballaz S., Morales I., Rodriguez M., Obeso J.A. (2013). Ascorbate prevents cell death from prolonged exposure to glutamate in an *in vitro* model of human dopaminergic neurons. J. Neurosci. Res..

[20] Dorner J.L., Miller B.R., Klein E.L., Murphy-Nakhnikian A., Andrews R.L., Barton S.J., Rebec G.V. (2009). Corticostriatal dysfunction underlies diminished striatal ascorbate release in the R6/2 mouse model of Huntington’s disease. Brain Res..

[21] Rebec G.V., Pierce R.C. (1994). A vitamin as neuromodulator: Ascorbate release into the extracellular fluid of the brain regulates dopaminergic and glutamatergic transmission. Prog. Neurobiol..

[22] Rebec G.V., Barton S.J., Marseilles A.M., Collins K. (2003). Ascorbate treatment attenuates the Huntington behavioral phenotype in mice. Neuroreport.

[23] Spector R., Johanson C.E. (2014). The nexus of vitamin homeostasis and DNA synthesis and modification in mammalian brain. Mol. Brain.

[24] Blaschke K., Ebata K.T., Karimi M.M., Zepeda-Martinez J.A., Goyal P., Mahapatra S., Tam A., Laird D.J., Hirst M., Rao A. (2013). Vitamin C induces Tet-dependent DNA demethylation and a blastocyst-like state in ES cells. Nature.

[25] Yin R., Mao S.Q., Zhao B., Chong Z., Yang Y., Zhao C., Zhang D., Huang H., Gao J., Li Z. (2013). Ascorbic acid enhances Tet-mediated 5-methylcytosine oxidation and promotes DNA demethylation in mammals. J. Am. Chem. Soc..

[26] Minor E.A., Court B.L., Young J.I., Wang G. (2013). Ascorbate induces ten-eleven translocation (Tet) methylcytosine dioxygenase-mediated generation of 5-hydroxymethylcytosine. J. Biol. Chem..

[27] Rudenko A., Dawlaty M.M., Seo J., Cheng A.W., Meng J., Le T., Faull K.F., Jaenisch R., Tsai L.H. (2013). Tet1 is critical for neuronal activity-regulated gene expression and memory extinction. Neuron.

[28] Meister A. (1994). Glutathione, ascorbate, and cellular protection. Cancer Res..

[29] Smuda D.G.M. (2013). Maillard degradation pathways of vitamin C. Angew. Chem. Int. Ed..

[30] Fan X., Reneker L.W., Obrenovich M.E., Strauch C., Cheng R., Jarvis S.M., Ortwerth B.J., Monnier V.M. (2006). Vitamin C mediates chemical aging of lens crystallins by the Maillard reaction in a humanized mouse model. Proc. Natl. Acad. Sci. USA.

[31] Carr A., Frei B. (1999). Does vitamin C act as a pro-oxidant under physiological conditions?. FASEB J..

[32] Halliwell B. (1996). Vitamin C: Antioxidant or pro-oxidant *in vivo*?. Free Radic. Res..

[33] Premkumar K., Bowlus C.L. (2004). Ascorbic acid does not increase the oxidative stress induced by dietary iron in C3H mice. J. Nutr..

[34] Collis C.S., Yang M., Diplock A.T., Hallinan T., Rice-Evans C.A. (1997). Effects of co-supplementation of iron with ascorbic acid on antioxidant—Pro-oxidant balance in the guinea pig. Free Radic. Res..

[35] Gerster H. (1999). High-dose vitamin C: A risk for persons with high iron stores?. Int. J. Vitam Nutr. Res..

[36] Loef M., Walach H. (2012). Copper and iron in Alzheimer’s disease: A systematic review and its dietary implications. Br. J. Nutr..

[37] Crespo A.C., Silva B., Marques L., Marcelino E., Maruta C., Costa S., Timoteo A., Vilares A., Couto F.S., Faustino P. (2014). Genetic and biochemical markers in patients with Alzheimer’s disease support a concerted systemic iron homeostasis dysregulation. Neurobiol. Aging.

[38] Sinha M., Bhowmick P., Banerjee A., Chakrabarti S. (2013). Antioxidant role of amyloid beta protein in cell-free and biological systems: Implication for the pathogenesis of Alzheimer disease. Free Radic. Biol. Med..

[39] Hammarstrom L. (1966). Autoradiographic studies on the distribution of C14-labelled ascorbic acid and dehydroascorbic acid. Acta Physiol. Scand..

[40] Spector R., Lorenzo A.V. (1973). Ascorbic acid homeostasis in the central nervous system. Am. J. Physiol..

[41] Savini I., Rossi A., Pierro C., Avigliano L., Catani M.V. (2008). SVCT1 and SVCT2: Key proteins for vitamin C uptake. Amino Acids.

[42] Tsukaguchi H., Tokui T., Mackenzie B., Berger U.V., Chen X.Z., Wang Y., Brubaker R.F., Hediger M.A. (1999). A family of mammalian Na+-dependent L-ascorbic acid transporters. Nature.

[43] Wang Y., Mackenzie B., Tsukaguchi H., Weremowicz S., Morton C.C., Hediger M.A. (2000). Human vitamin C (l-ascorbic acid) transporter SVCT1. Biochem. Biophys. Res. Commun..

[44] Lindblad M., Tveden-Nyborg P., Lykkesfeldt J. (2013). Regulation of Vitamin C Homeostasis during Deficiency. Nutrients.

[45] Erichsen H.C., Eck P., Levine M., Chanock S. (2001). Characterization of the genomic structure of the human vitamin C transporter SVCT1 (SLC23A2). J. Nutr..

[46] Erichsen H.C., Peters U., Eck P., Welch R., Schoen R.E., Yeager M., Levine M., Hayes R.B., Chanock S. (2008). Genetic variation in sodium-dependent vitamin C transporters SLC23A1 and SLC23A2 and risk of advanced colorectal adenoma. Nutr. Cancer.

[47] Michels A.J., Hagen T.M., Frei B. (2013). Human genetic variation influences vitamin C homeostasis by altering vitamin C transport and antioxidant enzyme function. Annu. Rev. Nutr..

[48] Eck P., Erichsen H.C., Taylor J.G., Corpe C., Chanock S.J., Levine M. (2007). Genomic and functional analysis of the sodium-dependent vitamin C transporter SLC23A1-SVCT1. Genes Nutr..

[49] Corpe C.P., Tu H., Eck P., Wang J., Faulhaber-Walter R., Schnermann J., Margolis S., Padayatty S., Sun H., Wang Y. (2010). Vitamin C transporter Slc23a1 links renal reabsorption, vitamin C tissue accumulation, and perinatal survival in mice. J. Clin. Invest..

[50] Hierro C., Monte M.J., Lozano E., Gonzalez-Sanchez E., Marin J.J., Macias R.I. (2014). Liver metabolic/oxidative stress induces hepatic and extrahepatic changes in the expression of the vitamin C transporters SVCT1 and SVCT2. Eur. J. Nutr..

[51] Gess B., Sevimli S., Strecker J.K., Young P., Schabitz W.R. (2011). Sodium-dependent vitamin C transporter 2 (SVCT2) expression and activity in brain capillary endothelial cells after transient ischemia in mice. PLoS One.

[52] May J.M., Qu Z.C. (2010). Ascorbic acid prevents increased endothelial permeability caused by oxidized low density lipoprotein. Free Radic. Res..

[53] May J.M., Qu Z.C. (2011). Ascorbic acid prevents oxidant-induced increases in endothelial permeability. Biofactors.

[54] Amano A., Aigaki T., Maruyama N., Ishigami A. (2010). Ascorbic acid depletion enhances expression of the sodium-dependent vitamin C transporters, SVCT1 and SVCT2, and uptake of ascorbic acid in livers of SMP30/GNL knockout mice. Arch. Biochem. Biophys..

[55] Meredith M.E., Harrison F.E., May J.M. (2011). Differential regulation of the ascorbic acid transporter SVCT2 during development and in response to ascorbic acid depletion. Biochem. Biophys. Res. Commun..

[56] Nualart F., Castro T., Low M., Henriquez J.P., Oyarce K., Cisternas P., Garcia A., Yanez A.J., Bertinat R., Montecinos V.P. (2013). Dynamic expression of the sodium-vitamin C co-transporters, SVCT1 and SVCT2, during perinatal kidney development. Histochem. Cell Biol..

[57] Jimenez-Fernandez E., Ponce M., Zuasti E., Fernandez-Diaz C., Manchado M., Infante C. (2012). Molecular characterization and transcriptional regulation of the sodium-dependent vitamin C transporter genes (slc23a1 and slc23a2) in a teleost fish, the Senegalese sole (Solea senegalensis). Comp. Biochem. Physiol. Part B Biochem. Mol. Biol..

[58] Gess B., Lohmann C., Halfter H., Young P. (2010). Sodium-dependent vitamin C transporter 2 (SVCT2) is necessary for the uptake of l-ascorbic acid into Schwann cells. Glia.

[59] Maulen N.P., Henriquez E.A., Kempe S., Carcamo J.G., Schmid-Kotsas A., Bachem M., Grunert A., Bustamante M.E., Nualart F., Vera J.C. (2003). Up-regulation and polarized expression of the sodium-ascorbic acid transporter SVCT1 in post-confluent differentiated CaCo-2 cells. J. Biol. Chem..

[60] Boyer J.C., Campbell C.E., Sigurdson W.J., Kuo S.M. (2005). Polarized localization of vitamin C transporters, SVCT1 and SVCT2, in epithelial cells. Biochem. Biophys. Res. Commun..

[61] MacDonald L., Thumser A.E., Sharp P. (2002). Decreased expression of the vitamin C transporter SVCT1 by ascorbic acid in a human intestinal epithelial cell line. Br. J. Nutr..

[62] Varma S., Sobey K., Campbell C.E., Kuo S.M. (2009). Hierarchal contribution of N- and C-terminal sequences to the differential localization of homologous sodium-dependent vitamin C transporters, SVCT1 and SVCT2, in epithelial cells. Biochemistry.

[63] May J.M., Li L., Qu Z.C. (2010). Oxidized LDL up-regulates the ascorbic acid transporter SVCT2 in endothelial cells. Mol. Cell. Biochem..

[64] Qiao H., May J.M. (2008). Development of ascorbate transporters in brain cortical capillary endothelial cells in culture. Brain Res..

[65] Chothe P.P., Chutkan N., Sangani R., Wenger K.H., Prasad P.D., Thangaraju M., Hamrick M.W., Isales C.M., Ganapathy V., Fulzele S. (2013). Sodium-coupled vitamin C transporter (SVCT2): expression, function, and regulation in intervertebral disc cells. Spine J..

[66] Berger U.V., Hediger M.A. (2000). The vitamin C transporter SVCT2 is expressed by astrocytes in culture but not *in situ*. Neuroreport.

[67] Korcok J., Yan R., Siushansian R., Dixon S.J., Wilson J.X. (2000). Sodium-ascorbate cotransport controls intracellular ascorbate concentration in primary astrocyte cultures expressing the SVCT2 transporter. Brain Res..

[68] Oke A.F., May L., Adams R.N. (1987). Ascorbic acid distribution patterns in human brain. A comparison with nonhuman mammalian species. Ann. N. Y. Acad. Sci..

[69] Mefford I.N., Oke A.F., Adams R.N. (1981). Regional distribution of ascorbate in human brain. Brain Res..

[70] Rice M.E., Russo-Menna I. (1998). Differential compartmentalization of brain ascorbate and glutathione between neurons and glia. Neuroscience.

[71] Caprile T., Salazar K., Astuya A., Cisternas P., Silva-Alvarez C., Montecinos H., Millan C., de Los Angeles Garcia M., Nualart F. (2009). The Na+-dependent L-ascorbic acid transporter SVCT2 expressed in brainstem cells, neurons, and neuroblastoma cells is inhibited by flavonoids. J. Neurochem..

[72] Qiu S., Li L., Weeber E.J., May J.M. (2007). Ascorbate transport by primary cultured neurons and its role in neuronal function and protection against excitotoxicity. J. Neurosci. Res..

[73] Mun G.H., Kim M.J., Lee J.H., Kim H.J., Chung Y.H., Chung Y.B., Kang J.S., Hwang Y.I., Oh S.H., Kim J.G. (2006). Immunohistochemical study of the distribution of sodium-dependent vitamin C transporters in adult rat brain. J. Neurosci. Res..

[74] Garcia Mde L., Salazar K., Millan C., Rodriguez F., Montecinos H., Caprile T., Silva C., Cortes C., Reinicke K., Vera J.C. (2005). Sodium vitamin C cotransporter SVCT2 is expressed in hypothalamic glial cells. Glia.

[75] Chinoy N.J., Sanjeevan A.G. (1978). On the specificity of alcoholic acidic silver nitrate reagent for the histochemical localization of ascorbic acid. A reappraisal. Histochemistry.

[76] Berger U.V., Lu X.C., Liu W., Tang Z., Slusher B.S., Hediger M.A. (2003). Effect of middle cerebral artery occlusion on mRNA expression for the sodium-coupled vitamin C transporter SVCT2 in rat brain. J. Neurochem..

[77] McHenry E.W., Reedman E.J., Sheppard M. (1938). The physiological properties of ascorbic acid: An effect upon the weights of guinea-pigs. Biochem. J..

[78] Svirbely J.L., Szent-Gyorgyi A. (1932). The chemical nature of vitamin C. Biochem. J..

[79] Szent-Gyorgyi A.H.W.N. (1933). “Hexuronic Acid” (Ascorbic Acid) as the Antiscorbutic Factor. Nature.

[80] Anderson WES A.H. (1924). The effect of acute scurvy on the subsequent nutrition and growth of guinea pigs. J. Biol. Chem..

[81] Burk R.F., Christensen J.M., Maguire M.J., Austin L.M., Whetsell W.O., May J.M., Hill K.E., Ebner F.F. (2006). A combined deficiency of vitamins E and C causes severe central nervous system damage in guinea pigs. J. Nutr..

[82] Hill K.E., Motley A.K., May J.M., Burk R.F. (2009). Combined selenium and vitamin C deficiency causes cell death in guinea pig skeletal muscle. Nutr. Res..

[83] Frikke-Schmidt H., Lykkesfeldt J. (2009). Role of marginal vitamin C deficiency in atherogenesis: *In vivo* models and clinical studies. Basic Clin. Pharmacol. Toxicol..

[84] Maeda N., Hagihara H., Nakata Y., Hiller S., Wilder J., Reddick R. (2000). Aortic wall damage in mice unable to synthesize ascorbic acid. Proc. Natl. Acad. Sci. USA.

[85] Harrison F.E., Meredith M.E., Dawes S.M., Saskowski J.L., May J.M. (2010). Low ascorbic acid and increased oxidative stress in gulo(−/−) mice during development. Brain Res..

[86] Harrison F.E., Yu S.S., Van Den Bossche K.L., Li L., May J.M., McDonald M.P. (2008). Elevated oxidative stress and sensorimotor deficits but normal cognition in mice that cannot synthesize ascorbic acid. J. Neurochem..

[87] Chen Y., Curran C.P., Nebert D.W., Patel K.V., Williams M.T., Vorhees C.V. (2012). Effect of vitamin C deficiency during postnatal development on adult behavior: functional phenotype of Gulo(−/−) knockout mice. Genes Brain Behav..

[88] Harrison F.E., May J.M., McDonald M.P. (2010). Vitamin C deficiency increases basal exploratory activity but decreases scopolamine-induced activity in APP/PSEN1 transgenic mice. Pharmacol. Biochem. Behav..

[89] Duggan G.E., Joan Miller B., Jirik F.R., Vogel H.J. (2011). Metabolic profiling of vitamin C deficiency in Gulo−/− mice using proton NMR spectroscopy. J. Biomol. NMR.

[90] Vissers M.C., Wilkie R.P. (2007). Ascorbate deficiency results in impaired neutrophil apoptosis and clearance and is associated with up-regulation of hypoxia-inducible factor 1alpha. J. Leukoc. Biol..

[91] Ishigami A., Fujita T., Handa S., Shirasawa T., Koseki H., Kitamura T., Enomoto N., Sato N., Shimosawa T., Maruyama N. (2002). Senescence marker protein-30 knockout mouse liver is highly susceptible to tumor necrosis factor-alpha- and Fas-mediated apoptosis. Am. J. Pathol..

[92] Kondo Y., Inai Y., Sato Y., Handa S., Kubo S., Shimokado K., Goto S., Nishikimi M., Maruyama N., Ishigami A. (2006). Senescence marker protein 30 functions as gluconolactonase in l-ascorbic acid biosynthesis, and its knockout mice are prone to scurvy. Proc. Natl. Acad. Sci. USA.

[93] Kondo Y., Sasaki T., Sato Y., Amano A., Aizawa S., Iwama M., Handa S., Shimada N., Fukuda M., Akita M. (2008). Vitamin C depletion increases superoxide generation in brains of SMP30/GNL knockout mice. Biochem. Biophys. Res. Commun..

[94] Son T.G., Zou Y., Jung K.J., Yu B.P., Ishigami A., Maruyama N., Lee J. (2006). SMP30 deficiency causes increased oxidative stress in brain. Mech. Ageing Dev..

[95] Beamer W.G., Rosen C.J., Bronson R.T., Gu W., Donahue L.R., Baylink D.J., Richardson C.C., Crawford G.C., Barker J.E. (2000). Spontaneous fracture (sfx): A mouse genetic model of defective peripubertal bone formation. Bone.

[96] Mohan S., Kapoor A., Singgih A., Zhang Z., Taylor T., Yu H., Chadwick R.B., Chung Y.S., Donahue L.R., Rosen C. (2005). Spontaneous fractures in the mouse mutant sfx are caused by deletion of the gulonolactone oxidase gene, causing vitamin C deficiency. J. Bone Miner. Res..

[97] Jiao Y., Zhang J., Yan J., Stuart J., Gibson G., Lu L., Willaims R., Wang Y.J., Gu W. (2011). Differential gene expression between wild-type and Gulo-deficient mice supplied with vitamin C. Genet. Mol. Biol..

[98] Ward M.S., Lamb J., May J.M., Harrison F.E. (2013). Behavioral and monoamine changes following severe vitamin C deficiency. J. Neurochem..

[99] Amano A., Tsunoda M., Aigaki T., Maruyama N., Ishigami A. (2014). Effect of ascorbic acid deficiency on catecholamine synthesis in adrenal glands of SMP30/GNL knockout mice. Eur. J. Nutr..

[100] Sotiriou S., Gispert S., Cheng J., Wang Y., Chen A., Hoogstraten-Miller S., Miller G.F., Kwon O., Levine M., Guttentag S.H. (2002). Ascorbic-acid transporter Slc23a1 is essential for vitamin C transport into the brain and for perinatal survival. Nat. Med..

[101] Harrison F.E., Dawes S.M., Meredith M.E., Babaev V.R., Li L., May J.M. (2010). Low vitamin C and increased oxidative stress and cell death in mice that lack the sodium-dependent vitamin C transporter SVCT2. Free Radic. Biol. Med..

[102] Bornstein S.R., Yoshida-Hiroi M., Sotiriou S., Levine M., Hartwig H.G., Nussbaum R.L., Eisenhofer G. (2003). Impaired adrenal catecholamine system function in mice with deficiency of the ascorbic acid transporter (SVCT2). FASEB J..

[103] Meredith M.E., May J.M. (2013). Regulation of embryonic neurotransmitter and tyrosine hydroxylase protein levels by ascorbic acid. Brain Res..

[104] Harrison F.E., Best J.L., Meredith M.E., Gamlin C.R., Borza D.B., May J.M. (2012). Increased expression of SVCT2 in a new mouse model raises ascorbic acid in tissues and protects against paraquat-induced oxidative damage in lung. PLoS One.

[105] Babaev V.R., Whitesell R.R., Li L., Linton M.F., Fazio S., May J.M. (2011). Selective macrophage ascorbate deficiency suppresses early atherosclerosis. Free Radic. Biol. Med..

[106] Babaev V.R., Li L., Shah S., Fazio S., Linton M.F., May J.M. (2010). Combined vitamin C and vitamin E deficiency worsens early atherosclerosis in apolipoprotein E-deficient mice. Arterioscler. Thromb. Vasc. Biol..

[107] Kook S.Y., Lee K.M., Kim Y., Cha M.Y., Kang S., Baik S.H., Lee H., Park R., Mook-Jung I. (2014). High-dose of vitamin C supplementation reduces amyloid plaque burden and ameliorates pathological changes in the brain of 5XFAD mice. Cell. Death Dis..

[108] Pierce M.R., Diasio D.L., Rodrigues L.M., Harrison F.E., May J.M. (2013). Combined vitamin C and E deficiency induces motor defects in gulo(−/−)/SVCT2(+/−) mice. Nutr. Neurosci..

[109] Morris M.C., Beckett L.A., Scherr P.A., Hebert L.E., Bennett D.A., Field T.S., Evans D.A. (1998). Vitamin E and vitamin C supplement use and risk of incident Alzheimer disease. Alzheimer Dis. Assoc. Disord..

[110] Morris M.C., Evans D.A., Bienias J.L., Tangney C.C., Bennett D.A., Aggarwal N., Wilson R.S., Scherr P.A. (2002). Dietary intake of antioxidant nutrients and the risk of incident Alzheimer disease in a biracial community study. JAMA.

[111] Carr A.C., Bozonet S.M., Pullar J.M., Simcock J.W., Vissers M.C. (2013). A Randomized Steady-State Bioavailability Study of Synthetic *versus* Natural (Kiwifruit-Derived) Vitamin C. Nutrients.

[112] Gray S.L., Anderson M.L., Crane P.K., Breitner J.C., McCormick W., Bowen J.D., Teri L., Larson E. (2008). Antioxidant vitamin supplement use and risk of dementia or Alzheimer’s disease in older adults. J. Am. Geriatr. Soc..

[113] Luchsinger J.A., Tang M.X., Shea S., Mayeux R. (2003). Antioxidant vitamin intake and risk of Alzheimer disease. Arch. Neurol..

[114] Masaki K.H., Losonczy K.G., Izmirlian G., Foley D.J., Ross G.W., Petrovitch H., Havlik R., White L.R. (2000). Association of vitamin E and C supplement use with cognitive function and dementia in elderly men. Neurology.

[115] Zandi P.P., Anthony J.C., Khachaturian A.S., Stone S.V., Gustafson D., Tschanz J.T., Norton M.C., Welsh-Bohmer K.A., Breitner J.C. (2004). Reduced risk of Alzheimer disease in users of antioxidant vitamin supplements: the Cache County Study. Arch. Neurol..

[116] Grodstein F., Chen J., Willett W.C. (2003). High-dose antioxidant supplements and cognitive function in community-dwelling elderly women. Am. J. Clin. Nutr..

[117] Devore E.E., Kang J.H., Stampfer M.J., Grodstein F. (2013). The association of antioxidants and cognition in the Nurses' Health Study. Am. J. Epidemiol..

[118] Engelhart M.J., Geerlings M.I., Ruitenberg A., van Swieten J.C., Hofman A., Witteman J.C., Breteler M.M. (2002). Dietary intake of antioxidants and risk of Alzheimer disease. JAMA.

[119] Bowman G.L., Shannon J., Ho E., Traber M.G., Frei B., Oken B.S., Kaye J.A., Quinn J.F. (2011). Reliability and validity of food frequency questionnaire and nutrient biomarkers in elders with and without mild cognitive impairment. Alzheimer Dis. Assoc. Disord..

[120] Rodrigue K.M., Kennedy K.M., Devous M.D., Rieck J.R., Hebrank A.C., Diaz-Arrastia R., Mathews D., Park D.C. (2012). beta-Amyloid burden in healthy aging: Regional distribution and cognitive consequences. Neurology.

[121] Bowman G.L., Silbert L.C., Howieson D., Dodge H.H., Traber M.G., Frei B., Kaye J.A., Shannon J., Quinn J.F. (2012). Nutrient biomarker patterns, cognitive function, and MRI measures of brain aging. Neurology.

[122] Bowman G.L. (2012). Ascorbic acid, cognitive function, and Alzheimer’s disease: A current review and future direction. Biofactors.

[123] Goodwin J.S., Goodwin J.M., Garry P.J. (1983). Association between nutritional status and cognitive functioning in a healthy elderly population. JAMA.

[124] Gale C.R., Martyn C.N., Cooper C. (1996). Cognitive impairment and mortality in a cohort of elderly people. BMJ.

[125] Riviere S., Birlouez-Aragon I., Nourhashemi F., Vellas B. (1998). Low plasma vitamin C in Alzheimer patients despite an adequate diet. Int. J. Geriatr. Psychiatry.

[126] Charlton K.E., Rabinowitz T.L., Geffen L.N., Dhansay M.A. (2004). Lowered plasma vitamin C, but not vitamin E, concentrations in dementia patients. J. Nutr. Health Aging.

[127] Polidori M.C., Mecocci P. (2002). Plasma susceptibility to free radical-induced antioxidant consumption and lipid peroxidation is increased in very old subjects with Alzheimer disease. J. Alzheimer’s Dis. (JAD).

[128] Polidori M.C., Mattioli P., Aldred S., Cecchetti R., Stahl W., Griffiths H., Senin U., Sies H., Mecocci P. (2004). Plasma antioxidant status, immunoglobulin g oxidation and lipid peroxidation in demented patients: relevance to Alzheimer disease and vascular dementia. Dement. Geriatr. Cogn. Disord..

[129] Perrig W.J., Perrig P., Stahelin H.B. (1997). The relation between antioxidants and memory performance in the old and very old. J. Am. Geriatr. Soc..

[130] Spector R. (2009). Nutrient transport systems in brain: 40 years of progress. J. Neurochem..

[131] Paraskevas G.P., Kapaki E., Libitaki G., Zournas C., Segditsa I., Papageorgiou C. (1997). Ascorbate in healthy subjects, amyotrophic lateral sclerosis and Alzheimer’s disease. Acta Neurol. Scand..

[132] Quinn J., Suh J., Moore M.M., Kaye J., Frei B. (2003). Antioxidants in Alzheimer's disease-vitamin C delivery to a demanding brain. J. Alzheimers Dis..

[133] Glaso M., Nordbo G., Diep L., Bohmer T. (2004). Reduced concentrations of several vitamins in normal weight patients with late-onset dementia of the Alzheimer type without vascular disease. J. Nutr. Health Aging.

[134] Bowman G.L., Dodge H., Frei B., Calabrese C., Oken B.S., Kaye J.A., Quinn J.F. (2009). Ascorbic acid and rates of cognitive decline in Alzheimer's disease. J. Alzheimers Dis..

[135] Arlt S., Muller-Thomsen T., Beisiegel U.K., Ontush A. (2012). Effect of One-Year Vitamin C- and E-Supplementation on Cerebrospinal Fluid Oxidation Parameters and Clinical Course in Alzheimer’s Disease. Neurochem. Res..

[136] Galasko D.R., Peskind E., Clark C.M., Quinn J.F., Ringman J.M., Jicha G.A., Cotman C., Cottrell B., Montine T.J., Thomas R.G., Aisen P. (2012). Antioxidants for Alzheimer disease: A randomized clinical trial with cerebrospinal fluid biomarker measures. Arch. Neurol..

[137] Spector R., Johanson C.E. (2013). Sustained choroid plexus function in human elderly and Alzheimer’s disease patients. Fluids Barriers CNS.

[138] Polidori M.C., Pientka L. (2012). Bridging the pathophysiology of Alzheimer’s disease with vascular pathology: the feed-back, the feed-forward, and oxidative stress. J. Alzheimer’s Dis. (JAD).

[139] Polidori M.C., Pientka L., Mecocci P. (2012). A review of the major vascular risk factors related to Alzheimer’s disease. J. Alzheimer’s Dis. (JAD).

[140] de la Torre J.C. (2012). Cerebral hemodynamics and vascular risk factors: setting the stage for Alzheimer’s disease. J. Alzheimer’s Dis. (JAD).

[141] Aguero-Torres H., Kivipelto M., von Strauss E. (2006). Rethinking the dementia diagnoses in a population-based study: What is Alzheimer’s disease and what is vascular dementia? A study from the kungsholmen project. Dement. Geriatr. Cogn. Disord..

[142] de la Torre J.C., Stefano G.B. (2000). Evidence that Alzheimer’s disease is a microvascular disorder: The role of constitutive nitric oxide. Brain Res. Brain Res. Rev..

[143] Polidori M.C., Mecocci P., Frei B. (2001). Plasma vitamin C levels are decreased and correlated with brain damage in patients with intracranial hemorrhage or head trauma. Stroke.

[144] Knopman D., Boland L.L., Mosley T., Howard G., Liao D., Szklo M., McGovern P., Folsom A.R. (2001). Cardiovascular risk factors and cognitive decline in middle-aged adults. Neurology.

[145] Polidori M.C., Pratico D., Savino K., Rokach J., Stahl W., Mecocci P. (2004). Increased F2 isoprostane plasma levels in patients with congestive heart failure are correlated with antioxidant status and disease severity. J. Card. Fail..

[146] Polidori M.C., Pratico D., Ingegni T., Mariani E., Spazzafumo L., Del Sindaco P., Cecchetti R., Yao Y., Ricci S., Cherubini A. (2005). Effects of vitamin C and aspirin in ischemic stroke-related lipid peroxidation: Results of the AVASAS (Aspirin Versus Ascorbic acid plus Aspirin in Stroke) Study. Biofactors.

[147] Wendell C.R., Zonderman A.B., Metter E.J., Najjar S.S., Waldstein S.R. (2009). Carotid intimal medial thickness predicts cognitive decline among adults without clinical vascular disease. Stroke.

[148] Frei B. (2003). To C or not to C, that is the question!. J. Am. Coll. Cardiol..

[149] Polidori M.C., Mecocci P., Reimann A., Cherubini A., Cecchetti R., Briviba K., Stahl W., Sies H., Senin U. (1999). Plasma lipid peroxidation and vitamin C status in healthy centenarians. J. Am. Geriatr. Soc..

[150] Hunter D.C., Skinner M.A., Wolber F.M., Booth C.L., Loh J.M., Wohlers M., Stevenson L.M., Kruger M.C. (2012). Consumption of gold kiwifruit reduces severity and duration of selected upper respiratory tract infection symptoms and increases plasma vitamin C concentration in healthy older adults. Br. J. Nutr..

[151] Moretti M., Colla A., de Oliveira Balen G., dos Santos D.B., Budni J., de Freitas A.E., Farina M., Severo Rodrigues A.L. (2012). Ascorbic acid treatment, similarly to fluoxetine, reverses depressive-like behavior and brain oxidative damage induced by chronic unpredictable stress. J. Psychiatr. Res..

[152] Simon J.A., Hudes E.S., Tice J.A. (2001). Relation of serum ascorbic acid to mortality among US adults. J. Am. Coll. Nutr..

[153] Boekholdt S.M., Meuwese M.C., Day N.E., Luben R., Welch A., Wareham N.J., Khaw K.T. (2006). Plasma concentrations of ascorbic acid and C-reactive protein, and risk of future coronary artery disease, in apparently healthy men and women: the EPIC-Norfolk prospective population study. Br. J. Nutr..

[154] Khaw K.T., Bingham S., Welch A., Luben R., Wareham N., Oakes S., Day N. (2001). Relation between plasma ascorbic acid and mortality in men and women in EPIC-Norfolk prospective study: a prospective population study. European Prospective Investigation into Cancer and Nutrition. Lancet.

[155] Ellingsen I., Seljeflot I., Arnesen H., Tonstad S. (2009). Vitamin C consumption is associated with less progression in carotid intima media thickness in elderly men: A 3-year intervention study. Nutr. Metab. Cardiovasc. Dis. (NMCD).

[156] Hodis H.N., Mack W.J., LaBree L., Mahrer P.R., Sevanian A., Liu C.R., Liu C.H., Hwang J., Selzer R.H., Azen S.P. (2002). Alpha-tocopherol supplementation in healthy individuals reduces low-density lipoprotein oxidation but not atherosclerosis: The Vitamin E Atherosclerosis Prevention Study (VEAPS). Circulation.

[157] Zureik M., Galan P., Bertrais S., Mennen L., Czernichow S., Blacher J., Ducimetiere P., Hercberg S. (2004). Effects of long-term daily low-dose supplementation with antioxidant vitamins and minerals on structure and function of large arteries. Arterioscler. Thromb. Vasc. Biol..

[158] Luzzi S., Vella L., Bartolini M., Provinciali L., Silvestrini M. (2010). Atherosclerosis in the evolution of Alzheimer’s disease: Can treatment reduce cognitive decline?. J. Alzheimer’s Dis. (JAD).

[159] Terpstra M., Marjanska M., Henry P.G., Tkac I., Gruetter R. (2006). Detection of an antioxidant profile in the human brain *in vivo* via double editing with MEGA-PRESS. Magn. Reson. Med..

[160] Emir U.E., Raatz S., McPherson S., Hodges J.S., Torkelson C., Tawfik P., White T., Terpstra M. (2011). Noninvasive quantification of ascorbate and glutathione concentration in the elderly human brain. NMR Biomed..

